# WNP: A Novel Algorithm for Gene Products Annotation from Weighted Functional Networks

**DOI:** 10.1371/journal.pone.0038767

**Published:** 2012-06-28

**Authors:** Alberto Magi, Lorenzo Tattini, Matteo Benelli, Betti Giusti, Rosanna Abbate, Stefano Ruffo

**Affiliations:** 1 Dipartimento di Area Critica Medico-Chirurgica, Università degli Studi di Firenze, Firenze, Italy; 2 Centro Interdipartimentale per lo Studio delle Dinamiche Complesse (CSDC), Università degli Studi di Firenze, Firenze, Italy; 3 Unità di Diagnostica Genetica, Dipartimento di Laboratorio, Azienda Ospedaliero-Universitaria Careggi, Firenze, Italy; 4 Dipartimento di Energetica Sergio Stecco, Università degli Studi di Firenze, Firenze, Italy; Technical University of Madrid, Italy

## Abstract

Predicting the biological function of all the genes of an organism is one of the fundamental goals of computational system biology. In the last decade, high-throughput experimental methods for studying the functional interactions between gene products (GPs) have been combined with computational approaches based on Bayesian networks for data integration. The result of these computational approaches is an interaction network with weighted links representing connectivity likelihood between two functionally related GPs. The weighted network generated by these computational approaches can be used to predict annotations for functionally uncharacterized GPs. Here we introduce Weighted Network Predictor (WNP), a novel algorithm for function prediction of biologically uncharacterized GPs. Tests conducted on simulated data show that WNP outperforms other 5 state-of-the-art methods in terms of both specificity and sensitivity and that it is able to better exploit and propagate the functional and topological information of the network. We apply our method to Saccharomyces cerevisiae yeast and Arabidopsis thaliana networks and we predict Gene Ontology function for about 500 and 10000 uncharacterized GPs respectively.

## Introduction

Understanding how an organism functions is a task that requires the knowledge of molecular, biochemical, cellular and phenotypic effects of all genes. Although high throughput technologies, such as microarray and new sequencing platforms, allow for monitoring the molecular activity of tens of thousands genes simultaneously, experimental evidence of gene functions have been proven for a small fraction of all known genes. For instance, only approximately 12 K (K = 1000) of the 29 K genes in mouse have experimental evidence supporting their functional annotation. For Caenorhabditis elegans experimental evidences have been demonstrated for about a third (∼7.5 K) of its ∼20 K genes and even the well-characterized Saccharomyces cerevisiae still has ∼1 K of its genes without functional annotation (on a total of ∼6000 genes). During the last decade, several experimental strategies to study the functional interaction between gene products (GPs) have been developed: yeast-two-hybrid (Y2H) techniques allow for the detection of binding interactions between proteins [1], [2], expression profiling enables the measurements of transcript coexpression [3], [4], synthetic lethality and synthetic rescue experiments discover genetic interactions [5] while ChIP-Chip [6] and ChIP-seq [7] identify protein-DNA interactions. Although these high-throughput experimental strategies allow for the detection of thousands of interactions simultaneously, it is very difficult to extract biologically relevant relationships from noise within a single experiment. Moreover, no single experimental method can assess all the interactions in the interactome of an organism. To overcome the limits of single experiment analysis and to construct global networks of functional relationships, computational approaches have been developed for integrating data from multiple, often unrelated, proteomics and genomics experiments. The integration of multiple types of genomic data has been shown to be much more sensitive with respect to single datasets in the detection of functional relationships between genes, leading to “high-confidence” networks [8]. The great majority of these methods, introduced in the literature with the pioneering works of Jansen *et al.* [9], Lee *et al.* [10] and Troyanskaya *et al.* [11] are based on Bayesian network framework where many interaction experiments are used as features for a classifier of interactions/noninteractions. A probabilistic score is assigned to each possible interaction by training the classifier on a gold-standard set of true and false interactions. The output of these computational schemes is an interaction network where the links are represented by a score that measures the probability that two nodes are functionally related. Once the high-confidence weighted network is built, it can be used to predict annotations for uncharacterized GPs, such as GP function or localization [12]. The prediction of unannotated GPs is performed by means of prediction algorithms which may fall into two different categories: direct annotation schemes exploit neighbours’ functions for the annotation of a target GP while module-assisted schemes first cluster the network into modules of related GPs and then annotate each module according to the functions of its members. In this paper we will focus on direct methods. The key idea of all direct methods is that GPs interacting in a network are more likely to share the same biological function. Hishigaki *et al.* [13] introduced the first direct method for GP function prediction based on 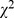 score: for each *p* GP they examine the *n*-neighbors, assigning a score 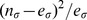 to each function 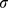, where 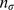 is the number of GPs in the *n*-neighbors of *p* with function 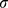 and 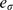 is the expected value of this number based on the frequency of 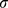 among the networks GPs. Nabieva *et al.* [14] introduced a flow-based approach: each annotated GP in the network is treated as a source of “functional flow”. First, functional flow spreading over time is calculated, then biological functions of uncharacterized GPs is predicted according to the flow they receive during the simulation. Chua *et al.* [15] devised a prediction algorithm that takes into account the relation between network distance and functional similarity. They studied the 1− and 2− neighborhoods of a target GP and proposed a functional score that weights links between GPs according to the inverse of their distance. Vazquez *et al.* [16] developed an optimization scheme assigning a function to each unannotated GP by maximizing the number of edges that connect GPs (unannotated or previously annotated) with the same functional category. While prediction algorithms by Chua *et al.* [15] and Nabieva *et al.* [14] exploit weighted links of probabilistic functional networks, the method devised by Vasquez *et al.* works on binary networks: the elements of the adjacency matrix can only take values of 0 (uncoupled nodes) and 1 (coupled nodes). In the present work we extend the annotation strategy proposed by Vazquez *et al.* [16] to exploit the weighted links of probabilistic functional networks (PFN). A novel algorithm, Weighted Network Predictor (WNP), for predicting the function of biologically uncharacterized GPs is presented. Testing WNP on simulated data we show that it outperforms other 5 state-of-the-art methods in terms of both specificity and sensitivity as it more efficently exploits and propagates the functional and topological information of the network. We apply our method to the PFNs of Saccharomyces cerevisiae and Arabidopsis thaliana and we predict the Gene Ontology (GO) [17] function for approximately 500 and 10000 uncharacterized GPs respectively.

## Materials and Methods

### Probabilstic Functional Networks

Probabilistic functional gene networks are built integrating heterogeneous genomics data. Data integration is performed exploiting the notion of “functional coupling” [10], [18]. The concept of functional coupling transcend the idea of physical interaction due to binding. GPs involved in a certain biological process may not show binding interactions. For instance, proteins involved in the same biological pathway, but in different biochemical steps, are functionally associated even in the absence of binding interactions. This concept of functional coupling is inclusive and allows for the integration of many different types of data capturing diverse types of associations (e.g., binding interactions, regulatory interactions, membership in the same protein complex, genetic interaction etc.). Exploiting the idea of functional coupling, Lee *et al.* [10], developed a Bayesian statistical method that allows for the evaluation of functional associations between GPs by integrating many heterogeneous functional data. The Bayesian approach is based on a Log Likelihood Score (LLS) that measures the likelihood of GPs pairs to be functionally associated on the basis of experimental data. Since the scores for each experiment are measured on a common benchmark, experiments are comparable and scores can be added to estimate the confidence of combined evidence. Once the scores of all the experimental data have been integrated, the probabilistic functional network – with the LLS measuring the probability of an interaction representing a true functional linkage between two GPs – is obtained. Scores greater than zero correspond to functional linkages, with higher scores indicating more confident connections. Thus, Lee and coworkers constructed PFN for organisms ranging from unicellular yeast [10], through invertebrate model organisms [19], to mammals [20]. In this paper we used the PFN of Saccharomyces cerevisiae YeastNet v.2 [21] and the PFN of Arabidopsis thaliana AraNet v.1 [22]. The YeastNet v.2 covers 102803 linkages among 5483 yeast proteins (95% of the validated proteome), while the AraNet v.1 covers 1062222 linkages among 19647 Arabidopsis proteins (73% of the validate proteome). The two PFNs were downloaded from http://www.yeastnet.org/.

### Prediction Scheme

In a pioneering work, Vazquez *et al.* [16] proposed to assign function 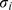 to each unannotated protein *i* of a Protein-Protein Interaction (PPI) network by maximizing the number of edges that connect proteins (unannotated or previously annotated) in the same functional category. The problem can be formulated as a global optimization task, where the scoring function *E* has to be maximized:
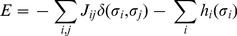
(1)where 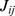 is the adjacency matrix of the interaction networks for uncharacterized GPs (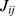 is equal to 1 if GP *i* and *j* interact and are uncharacterized, 0 otherwise), 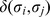 is the discrete delta function and 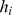 is the number of characterized GPs that link to GP *i* with function 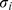. The first term of the score function represents the contribution of interactions between unannotated GPs while the second term refers to interactions between unannotated and previously annotated proteins. A simulated annealing optimization schedule was employed to maximize the total score and consequently to assign a biological function to each previously uncharacterized protein. Although this prediction scheme has the great advantage of using interaction with unannotated GPs, predicting GP function only for binary networks is a major drawback. Thus, in order to exploit the weighted structure of the PFNs, we extended the scoring function by Vazquez *et al.* [16] in the following manner:
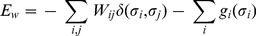
(2)where 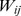 is the adjacency matrix of the interaction networks for uncharacterized GPs while 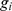 is the sum of the weights of edges linking GP i to characterized GPs with function 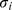. The extended version of the scoring function introduced by Vazquez allows for the prediction of unannotated GPs functions by maximizing the sum of LLS of edges that connect GPs (unannotated or previously annotated) with the same functional category. In order to minimize the Weighted Score 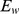 we used a minimization strategy based on the Generalized Simulated Annealing introduced by Tsallis and Stariolo [23] (see Section “Generalized Simulated Annealing” in Text S1).

### Functional Annotation

Gene Ontology [17] is a controlled and structured vocabulary made of a set of standard terms for the indexing and retrieving of information. The terms represent GP properties and cover three functional domains: cellular component (the parts of a cell or its extracellular environment), molecular function (the elemental activities of a gene product at the molecular level) and biological process (operations or sets of molecular events with a defined beginning and end, pertinent to the functioning of integrated living units: cells, tissues, organs, and organisms). Gene Ontology (GO) can be represented as a directed graph where nodes represent terms potentially connected by functional relationships. The graph structure of GO resemble a hierarchy where child terms are more specialized and parent terms are less specialized. Functional details can be tuned by cutting the GO structure at different hierarchic level. For the functional prediction of Saccharomyces cerevisiae and Arabidopsis thaliana we used “GO slim” – a subset of the terms in the whole GO. Go slims overview the ontology content without the details of the specific fine grained terms. The practice of associating the activities and localization of a gene product with GO terms (annotation) is carried out by curators such as the Saccharomyces Genome Database (SGD) for yeast and the Arabidopsis Information Resource (TAIR) for Arabidopsis thaliana. The GO slim version for yeast (downloaded from www.yeastgenome.org) contains 25 terms for cellular component, 25 terms for biological process and 45 terms for molecular function, while for Arabidopsis thaliana (downloaded from http://www.arabidopsis.org/) contains 16 terms for cellular component, 13 terms for biological process and 15 terms for molecular function.

### Performance Evaluation

The performance of function-prediction algorithms can be evaluated by means of two different approaches: leave-one-out and leave-a-percent-out cross-validation methods. Both are based on the same assumption: a certain fraction of GPs with known annotations is considered unannotated. In order to evaluate accuracy performances, the algorithm is applied to the unannotated GPs and the predictions on the selected GPs are then compared with the original annotations. The difference between the two cross-validation procedures consists in the amount of GPs to be cleared: with leave-one-out approaches the annotation of one GP at the time is cleared while with leave-a-percent-out methods the annotation of a certain percentage of GPs at the time is cleared. Since leave-one-out approaches are well suited for small dataset validation, we decided to evaluate the performance of WNP and compare it with other prediction algorithms by using a leave-a-percent-out criterion. Moreover, leave-a-percent-out approach fits better real-world annotation problems, where a large fraction of the genome/proteome is still unknown. Two different statistical measures were employed to study the prediction accuracy of WNP: the Area Under the Receiver Operating Characteristic Curve (AUC) and the success rate vs. functional degree curve. Receiver Operating Characteristic (ROC) curves were generated by plotting true positive rate (TPR) against false positive rate (FPR). TPR was calculated as the ratio between true positive (TP) prediction and total number of GPs to be predicted, while false positive rate (FPR) were determined as the ratio between false positive (FP) prediction and total number of GPs to be predicted. TP and FP are defined as the number of GPs correctly or incorrectly predicted [14]: if an algorithm assigns multiple predictions to an unknown GP, the latter is considered a TP if more than a half of the predicted functions are correct, otherwise it is marked as a FP. The couples of TPR/FPR (GPs correctly/incorrectly predicted) for different values of the algorithm thresholds allow for the construction of ROC-curves. To summarize ROC information content we calculated the relative AUC. In order to evaluate the performance of WNP and other algorithms in exploiting the functional topology of the weighted network for GP function prediction, we studied prediction success rate (SR) as a function of the functional degree (FD). The FD of a GP is the number of annotated GPs directly connected to the target GP. SR is defined as the ratio between the number of successful predictions against the total number of predictions. To build SR vs. FD curves we ranked, for each algorithm, all the functional predictions according to the algorithm score and we selected all the predictions with scores larger than a threshold. The threshold was selected for each simulation as the value that allows at least one prediction for each GP. SR vs. FD curves permit the estimation of the reliability of prediction algorithms as a function of the amount of information available for each GP in the network.

## Results

### Simulated Data Analysis

**Figure 1:**
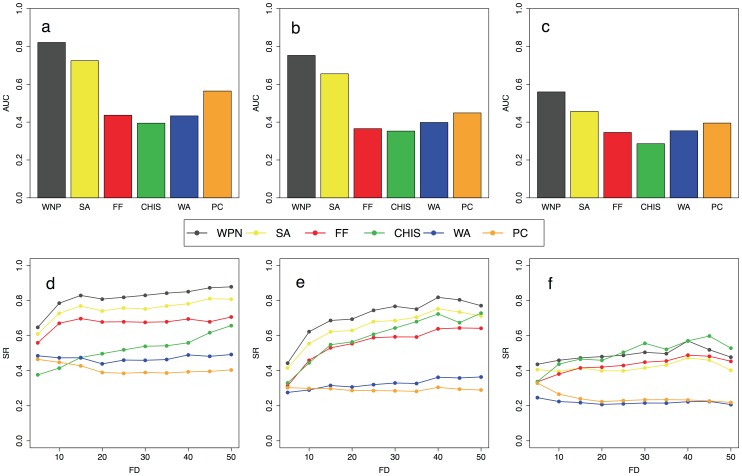
Comparison between function prediction algorithms for Saccharomyces cereviasiae. Six algorithms (WPN, SA, FF, WA, PC and CHI-Square) are compared with leave-a-percent-out criterion (see Section “Algorithm Comparison” in Text S1 for more details). For each algorithm the area under the ROC curve (AUC) and the SR vs. FD curves are averaged across all the leave-a-percent-out simulations we performed (5%, 10%, 15% and 20% of the annotated proteins cleared). The results are reported for the three categories of the GO database: cellular component (a, d), biological process (b, e) and molecular function (c, f).

**Figure 2:**
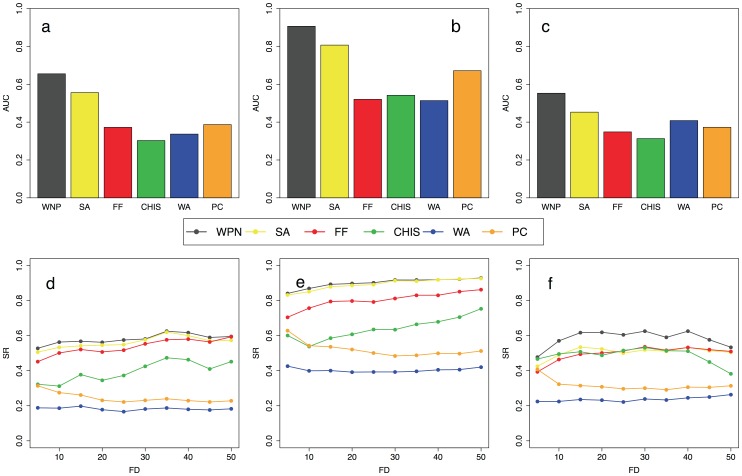
Comparison between function prediction algorithms for Arabidopsis thaliana. Six algorithms (WPN, SA, FF, WA, PC and CHI-Square) are compared with leave-a-percent-out criterion (see Section “Algorithm Comparison” in Text S1 for more details). For each algorithm the area under the ROC curve (AUC) and the SR vs. FD curves are averaged across all the leave-a-percent-out simulations we performed (5%, 10%, 15% and 20% of the annotated proteins cleared). The results are reported for the three categories of the GO database: cellular component (a, d), biological process (b, e) and molecular function (c, f).

To evaluate the ability of WNP in assigning Gene Ontology function to unannotated GPs we used the leave-a-percent-out strategy on the PFNs of Saccharomices cerevisiae and Arabidopsis thaliana and we compared the performance of our prediction scheme with other five state-of-the-art algorithms: the Simulated Annealing (SA) approach by Vazquez *et al.* [16], FunctionalFlow (FF) by Nabieva *et al.* [14], ChiSquare (CHIS) by Hishigaki *et al.* [13], the FS Weighted Averaging (WA) by Chua *et al.* [15] and the weighted average scheme (PC), again by Chua *et al.* [24] (see Section “Algorithm Comparison” in Text S1 for more details). Concerning yeast Saccharomyces cerevisiae we used the PFN YeastNet v2 inferred in Lee *et al.*, while for the Arabidopsis thaliana we used the PFN AraNet v1 inferred in Lee *et al.* (see Materials and Methods for more details). We performed the leave-a-percent-out validation by randomly removing the annotation of 5, 10, 15 and 20 percent of the annotated proteins for the three functional categories of Gene Ontology classification scheme (cellular component, biological process and molecular function). We applied the 6 prediction algorithms to 100 validation datasets for each ontology and the results of all these analyses are summarized in Figure 1 and Figures S1, S2, S3 and S4 for Saccharomyces cerevisiae and in Figure 2 and Figures S5, S6, S7 and S8 for Arabidopsis thaliana. The AUC barplots of Figures 1–2 and Figures S1, S2, S3, S4, S5, S6, S7 and S8 show that our prediction algorithm outperforms the other five state-of-the-art methods in terms of both sensitivity and specificity for all the three functional categories we used. The AUC barplots also show that the second best algorithm in terms of sensitivity/specificity tradeoff is the SA approach [16] followed by the PC method, the WA scheme and the FF algorithm. All these prediction methods achieve much better performance than the ChiSquare approach. The SR vs. FD curves (Figures 1d, 1e, 1f and 2d, 2e, 2f) show that the WNP algorithm obtains the best results also in terms of success rate independently by the functional information of the neighbour of each predicted protein. This is due to the fact that WNP algorithm is able to better exploit and propagate the functional and topological information of the network. The results reported in the SR vs. FD plots also show that the FF algorithm by Nebieva *et al.* and the Chi-square approach produce better results than the PC and WA algorithms of Chua *et al.* [15] The discrepancy between the performance measured by AUC and SR vs. FD analyses is mainly due to the fact that the prediction scores produced by the FF algorithm and Chi-square method are more informative of the score produced by the PC and WA algorithms of Chua *et al.* [15]. All the leave-a-percent-out validations we performed show that removing the annotation of 5, 10, 15 and 20 percent of the annotated GPs slightly affect the performance of our algorithm. For this reason, in order to study the prediction accuracy of our algorithm as a a function of the increasing number of cleared GPs, we extended the leave-a-percent-out cross validation up to removing the 90% of the annotated GPs. The results of these analyses are reported in Figure S9. Each plot of Figure S9 reports the global prediction SR as a function of the percentage of cleared annotated GPs. These results show that removing more than 50% of the annotated GPs drastically affects the performance of WPN algorithm, with the exception of the BP predictions made for the Arabidopsis thaliana. The weak dependence between WPN prediction accuracy and the percentage of cleared GPs for Arabidopsis thaliana BP ontology is due to the fact that a large proportion of the Arabidopsis thaliana GPs are annotated with the BP terms ‘other cellular processes’ and ‘other metabolic processes’: even when a large number of annotated GPs are removed, the WPN algorithm propagates these two terms in the network resulting in a large prediction accuracy as demonstrated in the plot of Figure S9.

### Functional Prediction of Uncharacterized Proteins

To test the real performance of the WNP algorithm in predicting the function of functionally uncharacterized proteins, we applied our global method to probabilistic functional networks of Saccharomyces cerevisiae and Arabidopsis thaliana. In order to asses the plausibility of our predictions we used the GO slim annotations made until january 2010 for yeast and GO slim annotations made until January 2011 for Arabidopsis, and we studied the overlap between our predictions and the annotations added to the GO database in the last months. A summary of the results of all the predictions is reported in Table 1. Moreover, we also looked for the informations related to predicted proteins in pubmed search. A list of all putative functional predictions made by WNP for Saccharomyces cerevisiae and Arabidopsis thaliana are provided in Tables S1 and S2 respectively.

**Table 1:** Summary of the prediction results obtained by WNP on the PFNs of Saccharomyces cerevisiae and Arabidopsis thaliana.

*Organism*	*Ontology*	*Predicted*	*Annotated*	*Matched*
	CC	680	94	29
SC	BP	1140	104	35
	MF	1840	99	45
	CC	10708	686	174
AT	BP	9996	2151	1492
	MF	8196	420	134

*Predicted* indicates the total number of GPs predicted by WPN. *Annotated* indicates the total number of GPs annotated by YGD and TAIR in the last N months for Saccharomyces cerevisiae (SC) and Arabidopsis thaliana (AT) respectively (N = 18 for Saccharomyces and N = 8 for Arabidopsis). *Matched* is the number of GPs annotated in the last N months that WNP correctly predicts.

### Saccharomyces Cerevisiae

**Figure 3:**
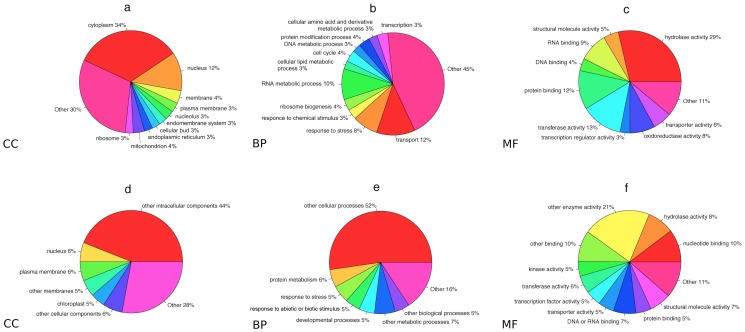
Prediction results for Saccharomyces cerevisiae and Arabidopsis thaliana. Pie charts report the distributions of the cellular component (a, d), biological process (b, e) and molecular function (c, f) terms predicted by the WNP algorithm. The results for Saccharomyces cerevisiae are shown in panels a, b and c. The results for Arabidopsis thaliana are reported in panels d, e and f.

The analyses performed by means of WNP on the yeast network allow us to annotate the Cellular Component (CC) of 680 previously uncharacterized proteins. Amongst all these prediction (see Figure 3), 34% of them fall under the cytoplasm category, 12% are part of the nucleus category and 4% are in membrane and mithocondrion categories. Considering Biological Process (BP) ontology, WNP predicted the annotation of 1140 proteins: about 12% of them fall into transport category, 10% belongs RNA metabolic process while 8% to stress category. Finally, for Molecular Function (MF) analysis our algorithm annotated 1840 functionally uncharacterized proteins: almost 30% of the predictions fall under the hydrolase activity, 13% are in transferase activity, 12% in protein binding category. To perform all these analyses we used the GO annotation made until January 2010. During 2009–2011, the Saccharomyces Genome Database associated slim terms to about 100 proteins in the YeastNet v2 network that were previously uncharacterized. Considering this set of novel annotations, our algorithm was able to correctly predict the MF category of 45 proteins, the BP of 35 proteins and the CC category of 29 proteins. Some examples of the capability of WNP in annotating Gene Ontology terms to uncharacterized proteins are reported in the following. WNP allowed for the prediction of the ‘nucleus’ localization of F-box protein DIA2/YOR080W and WSS1/YHR134W gene that were made by SGD curators according to Kile and Koepp [25] and van Heusden and Steensma [26] respectively. We were able to predict the annotation to ‘nucleolus’ term of the essential genes RRP36/YOR287C and GRC3/YLL035W that had been demonstrated to be nucleolar by Gérus *et al.* [27] and Braglia *et al.* [28] respectively. Concerning BP ontology, WNP predicted ‘ribosome biogenesis’ terms for UTP25/YIL091C and TSR4/YOL022C genes which are involved in ribosomal subunit maturation, ribosomal particle association, and ribosomal subunit nuclear export as reported by Li *et al.* [29]. Moreover, we associated the FDC1/YDR539W gene to the BP term ‘cellular aromatic compound metabolic process’. FDC1/YDR539W gene is essential for the decarboxylation of phenylacrylic acids in S. cerevisiae according to Mukai *et al.* [30]. Referring to MF categories we predicted the ‘protein binding’ term for the USA1/YML029W gene that functions as a major scaffold protein of the HRD-ligase [31]. Furthermore, WNP predicted the term ‘hydrolase activity’ for IMA2/YOL157C and PHM8/YER037W gene. IMA2 has been recently shown to encode a protein with alpha-glucosidase activity on isomaltose by Teste *et al.* [32] while overexpression of PHM8 in yeast resulted in an increase in the LPA phosphatase activity [33]. Finally the TRS120/YDR407C gene was predicted at ‘enzyme regulator activity’ and was manually annotated by the SGD at the ‘Rab guanyl-nucleotide exchange factor activity’ term by using the results obtained by Morozova *et al.* [34].

### Arabidopsis Thaliana

The WNP algorithm was able to predict the gene ontology annotation for about 10000 previously uncharacterized proteins (10708 proteins for CC, 9996 for BP and 8196 for MF). For CC ontology the 44% of the predictions fall under the ‘intacellular components category’, 6% under the ‘nucleus’ and ‘plasma membrane’ categories and 5% under ‘chloroplast’ category. Of all the 9996 biological process annotation made by our algorithm, around 50% belong to the other ‘cellular process’ category, 6% to ‘protein metabolism’ and 5% fall in the ‘response to stress’ and ‘developmental process’ categories. For molecular function ontology the 21% of all the annotations fall in the ‘other enzyme activity’, the 10% in ‘nucleotide binding’ and ‘other binding’ categories and the 8% in ‘hydrolase activity’ category. All the annotation made by WNP was performed by using go annotation made by the TAIR until january 2011. Since January 2011 the Arabidopsis Information Resource (TAIR) added the annotations of more than 2000, previously uncharacterized proteins for BP ontology, about 700 for CC ontology and about 400 for MF. By means of WNP algorithm we correctly predicted the CC term of 174 proteins, the MF of 134 proteins and the BP of about 1500 proteins which were functionally characterized in the last 8 months. In the following we report some of the results obtained with WNP. The ‘plasma membrane’ localization of the receptor kinase family gene CORYNE (CRN) was predicted in accordance with the work of Zhu *et al.* [35]. The authors showed that CRN was localized to the plasma membrane by means of fluorescence targeting. ‘Plasma membrane localization’ was predicted also for the receptor-like cytoplasmic kinase CAST AWAY which indeed interacts with HAE and EVR at the plasma membrane of Arabidopsis, as reported by Burr *et al.* [36]. WNP predicted the ‘nuclear localization’ for the PRP3 and ING2 proteins. Fujiwara *et* *al.* [37] and Lee *et al.* [38] confirmed respectively these results. Genes At3g03670 (putative peroxidase), At1g14540 (putative anionic peroxidase), and At1g14550 (putative anionic peroxidase) were annotated to the biological process term ‘response to stress’. All of them were demonstrated to be modulated by the transcription factor AtERF73/HRE1 during response to hypoxia in the work of Yang *et al.* [39]. TCP3, TCP10 and TCP24 genes - that were found implicated in leaf development by Efroni *et al.* [40] - were annotated to the ‘developmental processes’ term. Concerning MF annotation we were able to annotate the pPLAIII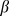 protein to the ontology term hydrolase activity according to the results of Li *et al.* [41]. The authors showed that pPLAIII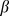 is responsible for phospholipids and galactolipids hydrolyses and additionally shows acyl-CoA thioesterase activity. Protein NRT1.9 was annotated to the ‘transporter activity’ term. Recently Wang *et al.* [42] have shown that NRT1.9 has a major role in phloem nitrate transport. Furthermore, WNP allowed to annotate the term ‘transporter activity’ to the AtAMT1;4 protein that was proven to be involved in transporting ammonium into pollen by Yuan *et al.* [43]. Finally, we were able to predict the ‘protein binding’ term for the PPI1 protein. Morandini *et al.* [44] demonstrated that PPI1 N-terminus is involved in the modulation of the PM H+-ATPase activity by binding to a site different from the 14-3-3 binding site and is located upstream of the trypsin cleavage site.

## Discussion

The development of computational methods for GPs function annotation based on interaction data is a challenging problem in bioinformatics. The combination of several sources of binary gene relationship data into a PFN is at present the best way to understand the complex structure of functional associations between elements of a cell. In this work, we extended the prediction approach proposed by Vazquez *et al.* [16] and we developed a novel algorithm (WNP) that is able to exploit the weighted nature of PFN for the global prediction of biological function of uncharacterized GPs. We have demonstrated the capability of WNP both in a cross validation setting and by closely examining its predictions over the complex PFNs of Saccharomices cerevisiae and Arabidopsis thaliana. By means of a leave-a-percent-out validation strategy we tested the prediction accuracy of our algorithm and we compared its performance with other 5 state-of-the-art prediction methods. The results of all these analyses clearly show that our method outperforms the others mentioned here in terms of both sensitivity and specificity. For yeast, the cellular localization of a GP was correctly predicted in about eight out of ten annotations, while for Arabidopsis thaliana the biological process in which the GP is involved in was correctly picked out in nine out of ten annotations. The validation analyses also show that our method performs better than the other methods in exploiting and propagating the functional and topological information of weighted protein interaction networks. As a further test, we studied the prediction capability of our algorithm in predicting the biological function of GPs that have been annotated in the last two years for both Saccharomices cerevisiae and Arabidopsis thaliana. Among ∼100 GPs annotated in 18 months for yeast, the WNP was able to correctly predict the MF category of 45 GPs, the BP of 35 GPs and the CC category of 29 proteins. For Arabidopsis thaliana the WNP correctly predicted the cellular component term of 174 proteins, the molecular function of 134 proteins and the biological process of about 1500 proteins that were functionally characterized in the last 8 months (2000 for BP, 700 for CC and 400 for MF). The current implementation of WPN takes into account only direct neighbours of uncharacterized GPs. At present we are extending the WPN to take into account level-2 and level-3 neighbours to improve its prediction capability.

## Supporting Information

Figure S1 **Comparison between Function prediction algorithms for Saccharomyces Cereviasiae.** Six algorithms (WPN, SA, FF, WA, PC and CHI-Square) are compared with leave-a-percent-out criterion for 5% of annotated GPs cleared. For each algorithm the area under the ROC curve (AUC) and the FD vs. SR curves are averaged across 100 simulations. The results are reported for the three categories of the GO database: cellular component (a, d), biological process (b, e) and molecular function (c, f).
(TIFF)

Figure S2 **Comparison between Function prediction algorithms for Saccharomyces Cereviasiae.** Six algorithms (WPN, SA, FF, WA, PC and CHI-Square) are compared with leave-a-percent-out criterion for 10% of annotated GPs cleared. For each algorithm the area under the ROC curve (AUC) and the FD vs. SR curves are averaged across 100 simulations. The results are reported for the three categories of the GO database: cellular component (a, d), biological process (b, e) and molecular function (c, f).
(TIFF)

Figure S3 **Comparison between Function prediction algorithms for Saccharomyces Cereviasiae.** Six algorithms (WPN, SA, FF, WA, PC and CHI-Square) are compared with leave-a-percent-out criterion for 15% of annotated GPs cleared. For each algorithm the area under the ROC curve (AUC) and the FD vs. SR curves are averaged across 100 simulations. The results are reported for the three categories of the GO database: cellular component (a, d), biological process (b, e) and molecular function (c, f).
(TIFF)

Figure S4 **Comparison between Function prediction algorithms for Saccharomyces Cereviasiae.** Six algorithms (WPN, SA, FF, WA, PC and CHI-Square) are compared with leave-a-percent-out criterion for 20% of annotated GPs cleared. For each algorithm the area under the ROC curve (AUC) and the FD vs. SR curves are averaged across 100 simulations. The results are reported for the three categories of the GO database: cellular component (a, d), biological process (b, e) and molecular function (c, f).
(TIFF)

Figure S5 **Comparison between Function prediction algorithms for Arabidopsis Thaliana.** Six algorithms (WPN, SA, FF, WA, PC and CHI-Square) are compared with leave-a-percent-out criterion for 5% of annotated GPs cleared. For each algorithm the area under the ROC curve (AUC) and the FD vs. SR curves are averaged across 100 simulations. The results are reported for the three categories of the GO database: cellular component (a, d), biological process (b, e) and molecular function (c, f).
(TIFF)

Figure S6 **Comparison between Function prediction algorithms for Arabidopsis Thaliana.** Six algorithms (WPN, SA, FF, WA, PC and CHI-Square) are compared with leave-a-percent-out criterion for 10% of annotated GPs cleared. For each algorithm the area under the ROC curve (AUC) and the FD vs. SR curves are averaged across 100 simulations. The results are reported for the three categories of the GO database: cellular component (a, d), biological process (b, e) and molecular function (c, f).
(TIFF)

Figure S7 **Comparison between Function prediction algorithms for Arabidopsis Thaliana.** Six algorithms (WPN, SA, FF, WA, PC and CHI-Square) are compared with leave-a-percent-out criterion for 15% of annotated GPs cleared. For each algorithm the area under the ROC curve (AUC) and the FD vs. SR curves are averaged across 100 simulations. The results are reported for the three categories of the GO database: cellular component (a, d), biological process (b, e) and molecular function (c, f).
(TIFF)

Figure S8 **Comparison between Function prediction algorithms for Arabidopsis Thaliana.** Six algorithms (WPN, SA, FF, WA, PC and CHI-Square) are compared with leave-a-percent-out criterion for 20% of annotated GPs cleared. For each algorithm the area under the ROC curve (AUC) and the FD vs. SR curves are averaged across 100 simulations. The results are reported for the three categories of the GO database: cellular component (a, d), biological process (b, e) and molecular function (c, f).
(TIFF)

Figure S9 **Prediction Success rate as a function of cleared GPs percentage.** The prediction accuracy of WPN algorithm is tested on leave-a-percent-out datasets with cleared annotated GPs that ranges between 10% and 90%. Each point represent the mean value of success rate across 100 simulations, while error bars are the standard deviation. The leave-a-percent-out validations were performed for Saccharomyces Cereviasiae (a, b, c) and Arabidopsis Thaliana (d, e, f). The results are reported for the three categories of the GO database: cellular component (a, d), biological process (b, e) and molecular function (c, f).
(TIFF)

Table S1 **A list of all putative functional predictions made by WNP for Saccharomyces cerevisiae.**
(XLS)

Table S2 **A list of all putative functional predictions made by WNP for Arabidopsis thaliana.**
(XLS)

Text S1 **Details concerning methods discussed in this work.**
(PDF)
