# Supplementary figures and images for: WNP: A Novel Algorithm for Gene Products Annotation from Weighted Functional Networks
Source: PLoS One. 2012 Jun 28;7(6):e38767. doi: 10.1371/journal.pone.0038767 (PMC3386258; doi:10.1371/journal.pone.0038767)

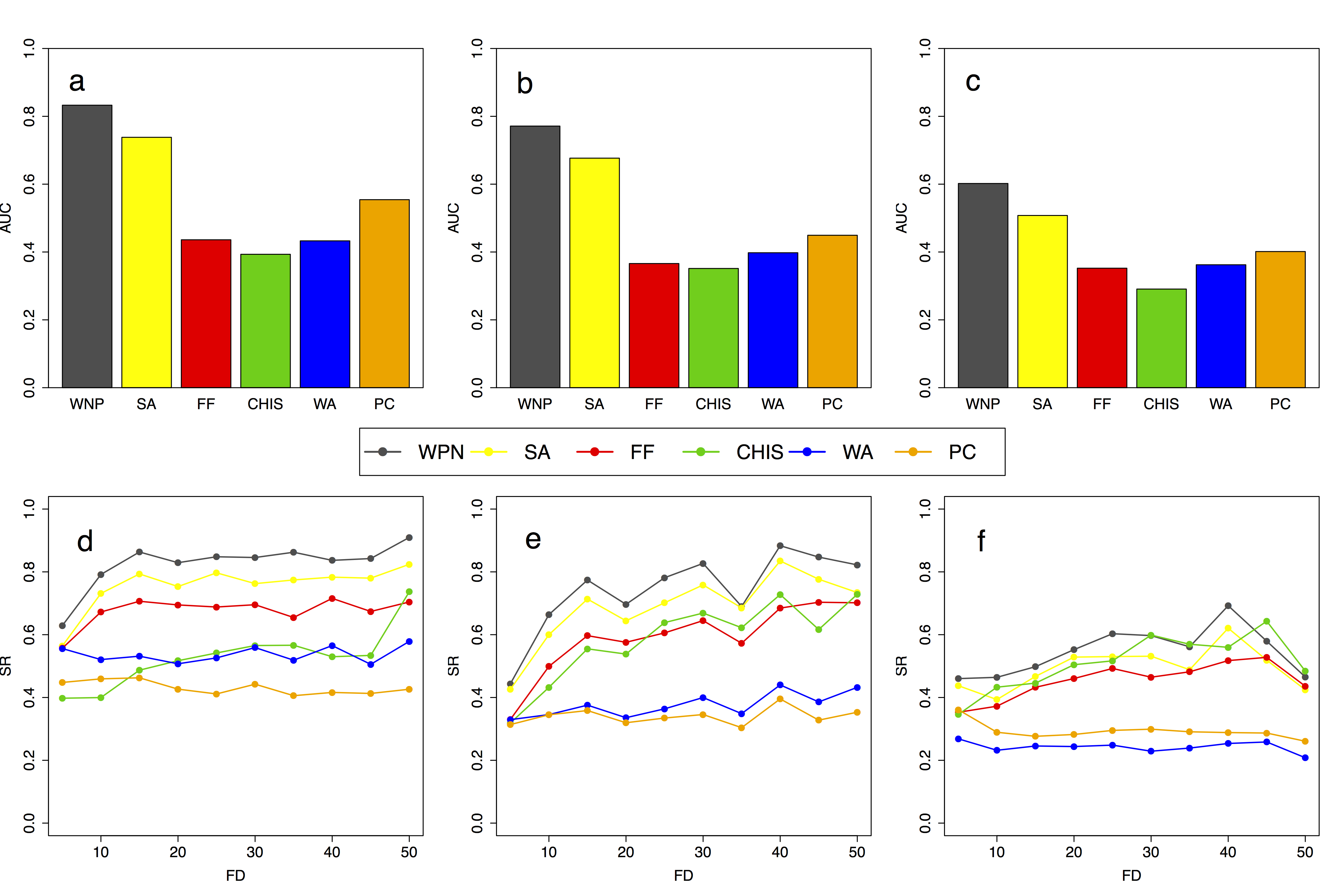

Supplement: Figure S1 — Comparison between Function prediction algorithms for Saccharomyces Cereviasiae. Six algorithms (WPN, SA, FF, WA, PC and CHI-Square) are compared with leave-a-percent-out criterion for 5% of annotated GPs cleared. For each algorithm the area under the ROC curve (AUC) and the FD vs. SR curves are averaged across 100 simulations. The results are reported for the three categories of the GO database: cellular component (a, d), biological process (b, e) and molecular function (c, f). (TIFF) [file pone.0038767.s001.tif]

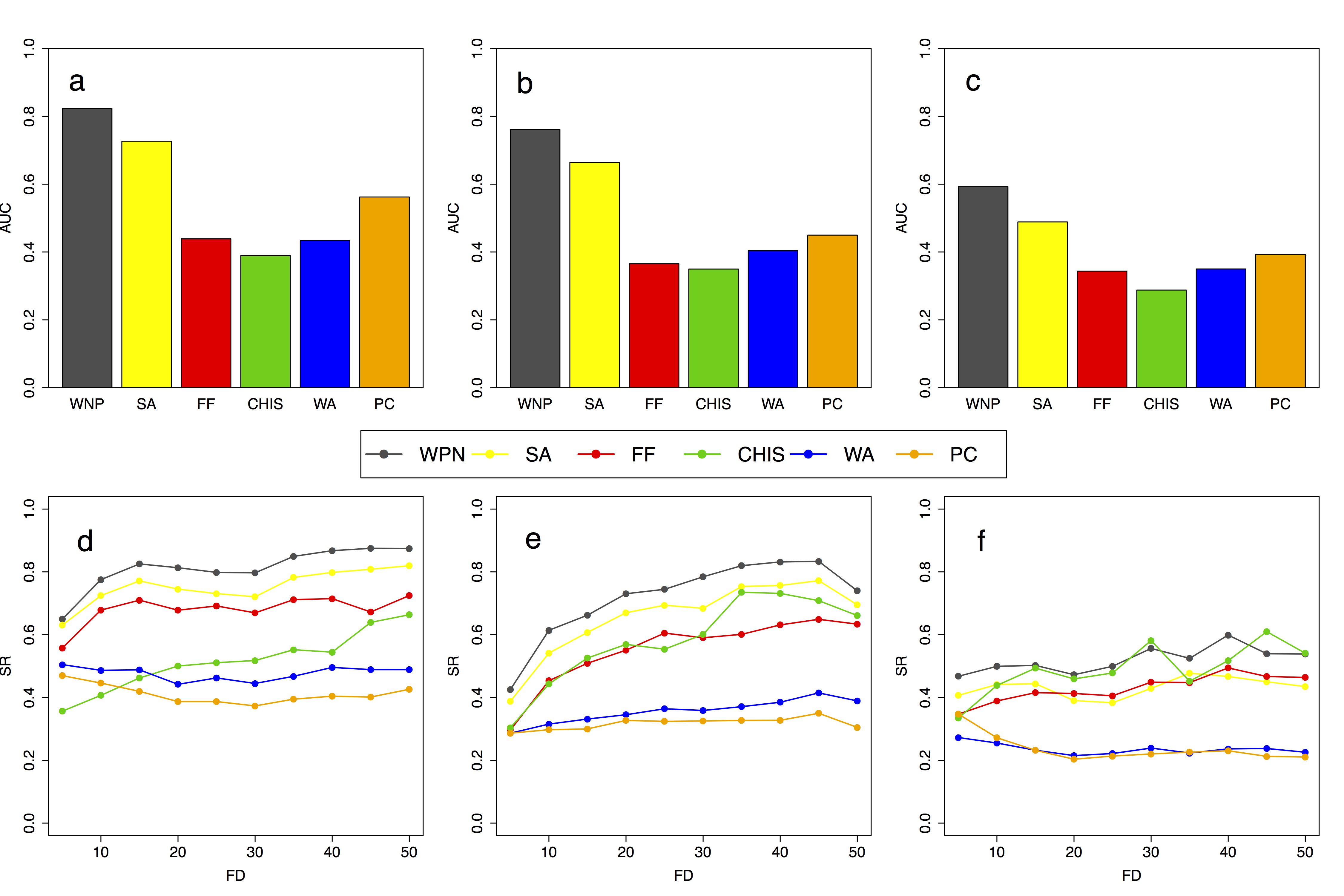

Supplement: Figure S2 — Comparison between Function prediction algorithms for Saccharomyces Cereviasiae. Six algorithms (WPN, SA, FF, WA, PC and CHI-Square) are compared with leave-a-percent-out criterion for 10% of annotated GPs cleared. For each algorithm the area under the ROC curve (AUC) and the FD vs. SR curves are averaged across 100 simulations. The results are reported for the three categories of the GO database: cellular component (a, d), biological process (b, e) and molecular function (c, f). (TIFF) [file pone.0038767.s002.tif]

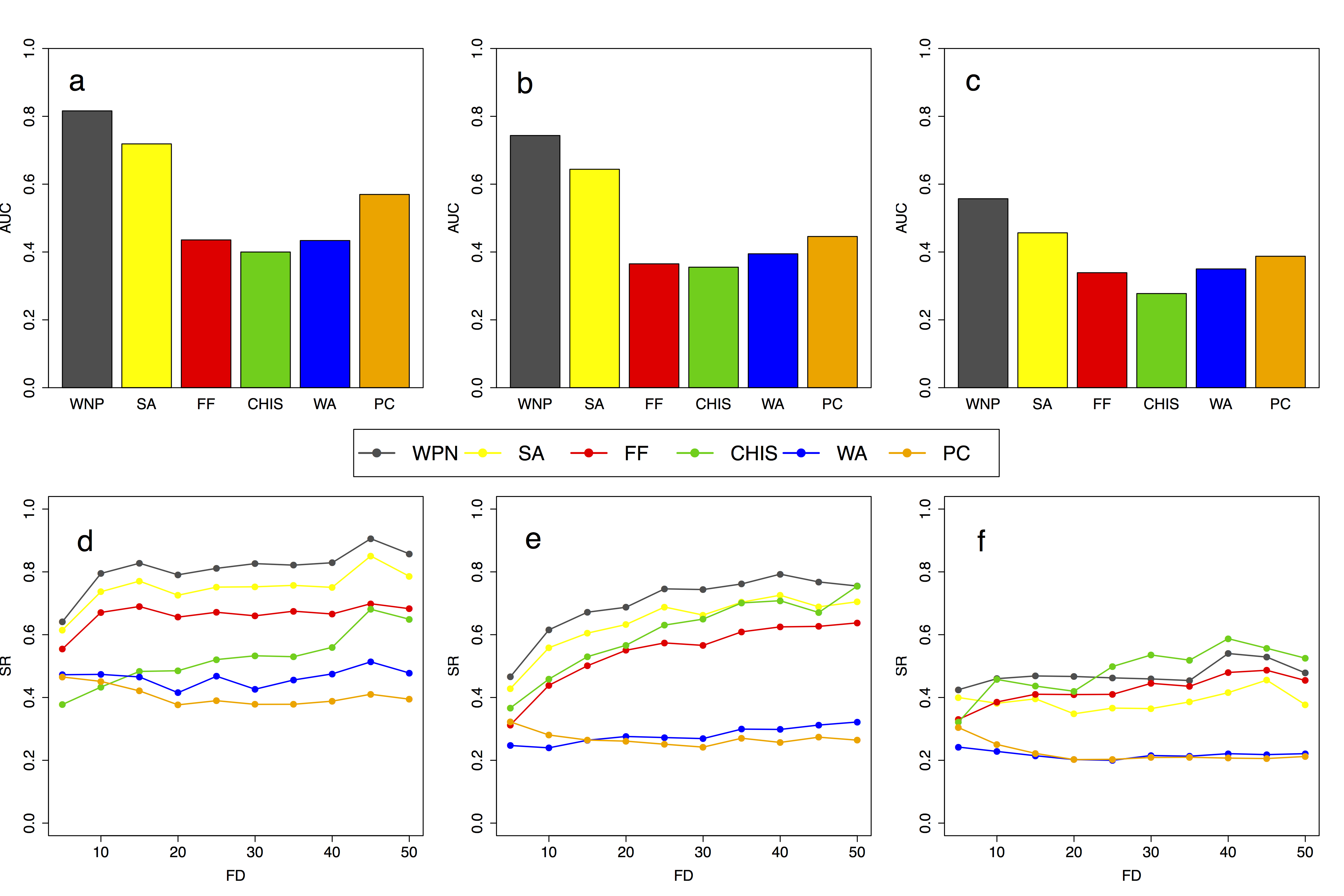

Supplement: Figure S3 — Comparison between Function prediction algorithms for Saccharomyces Cereviasiae. Six algorithms (WPN, SA, FF, WA, PC and CHI-Square) are compared with leave-a-percent-out criterion for 15% of annotated GPs cleared. For each algorithm the area under the ROC curve (AUC) and the FD vs. SR curves are averaged across 100 simulations. The results are reported for the three categories of the GO database: cellular component (a, d), biological process (b, e) and molecular function (c, f). (TIFF) [file pone.0038767.s003.tif]

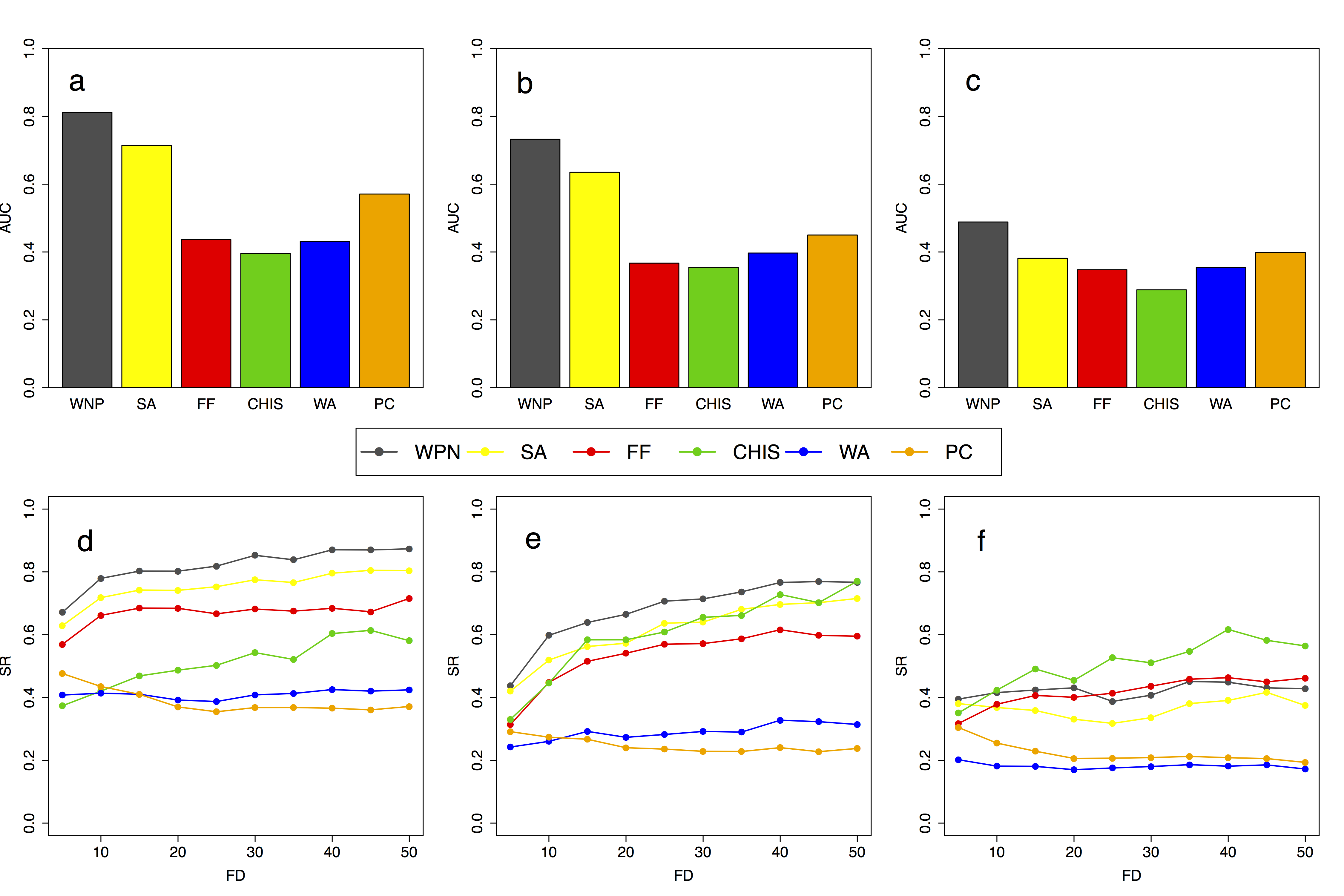

Supplement: Figure S4 — Comparison between Function prediction algorithms for Saccharomyces Cereviasiae. Six algorithms (WPN, SA, FF, WA, PC and CHI-Square) are compared with leave-a-percent-out criterion for 20% of annotated GPs cleared. For each algorithm the area under the ROC curve (AUC) and the FD vs. SR curves are averaged across 100 simulations. The results are reported for the three categories of the GO database: cellular component (a, d), biological process (b, e) and molecular function (c, f). (TIFF) [file pone.0038767.s004.tif]

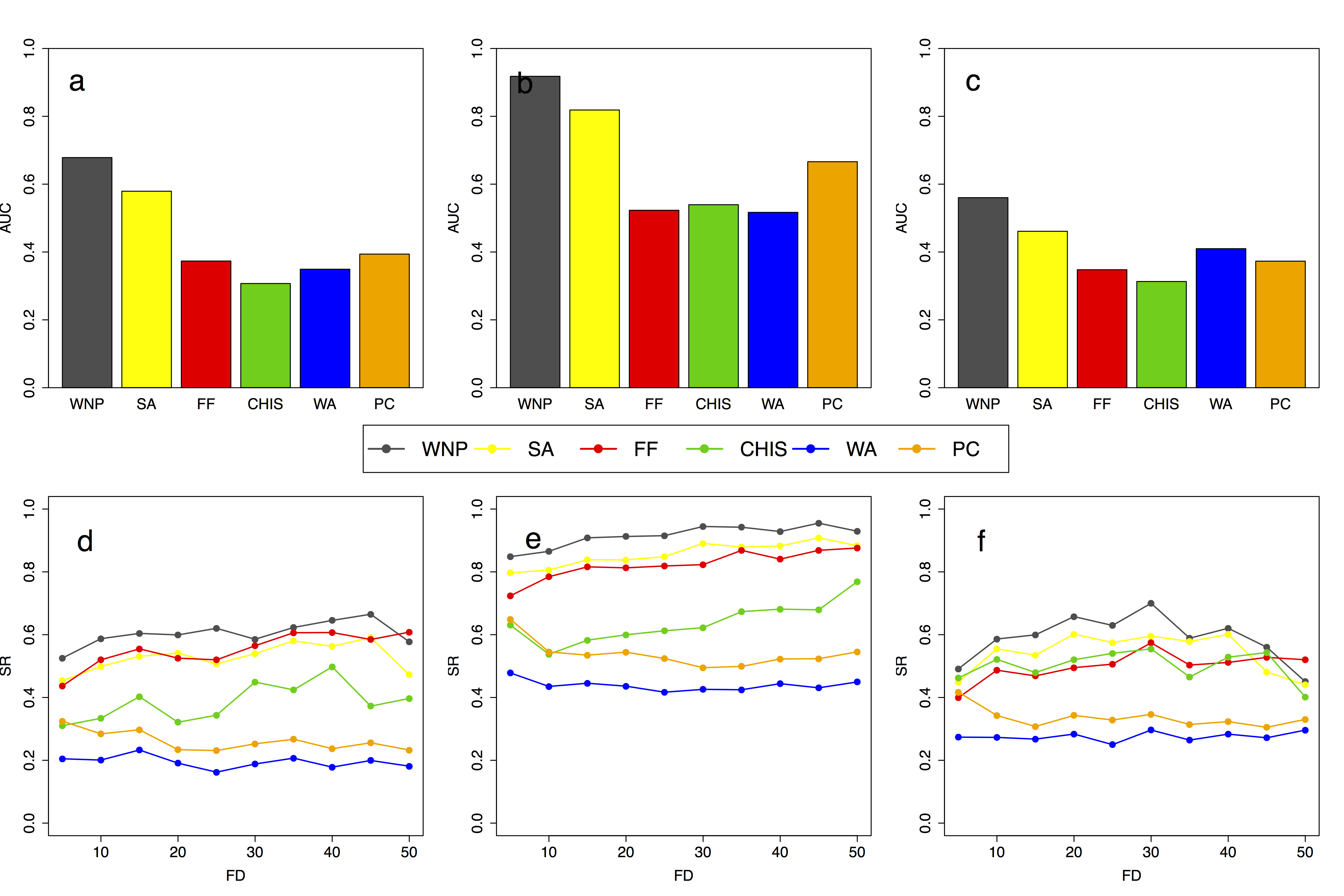

Supplement: Figure S5 — Comparison between Function prediction algorithms for Arabidopsis Thaliana. Six algorithms (WPN, SA, FF, WA, PC and CHI-Square) are compared with leave-a-percent-out criterion for 5% of annotated GPs cleared. For each algorithm the area under the ROC curve (AUC) and the FD vs. SR curves are averaged across 100 simulations. The results are reported for the three categories of the GO database: cellular component (a, d), biological process (b, e) and molecular function (c, f). (TIFF) [file pone.0038767.s005.tif]

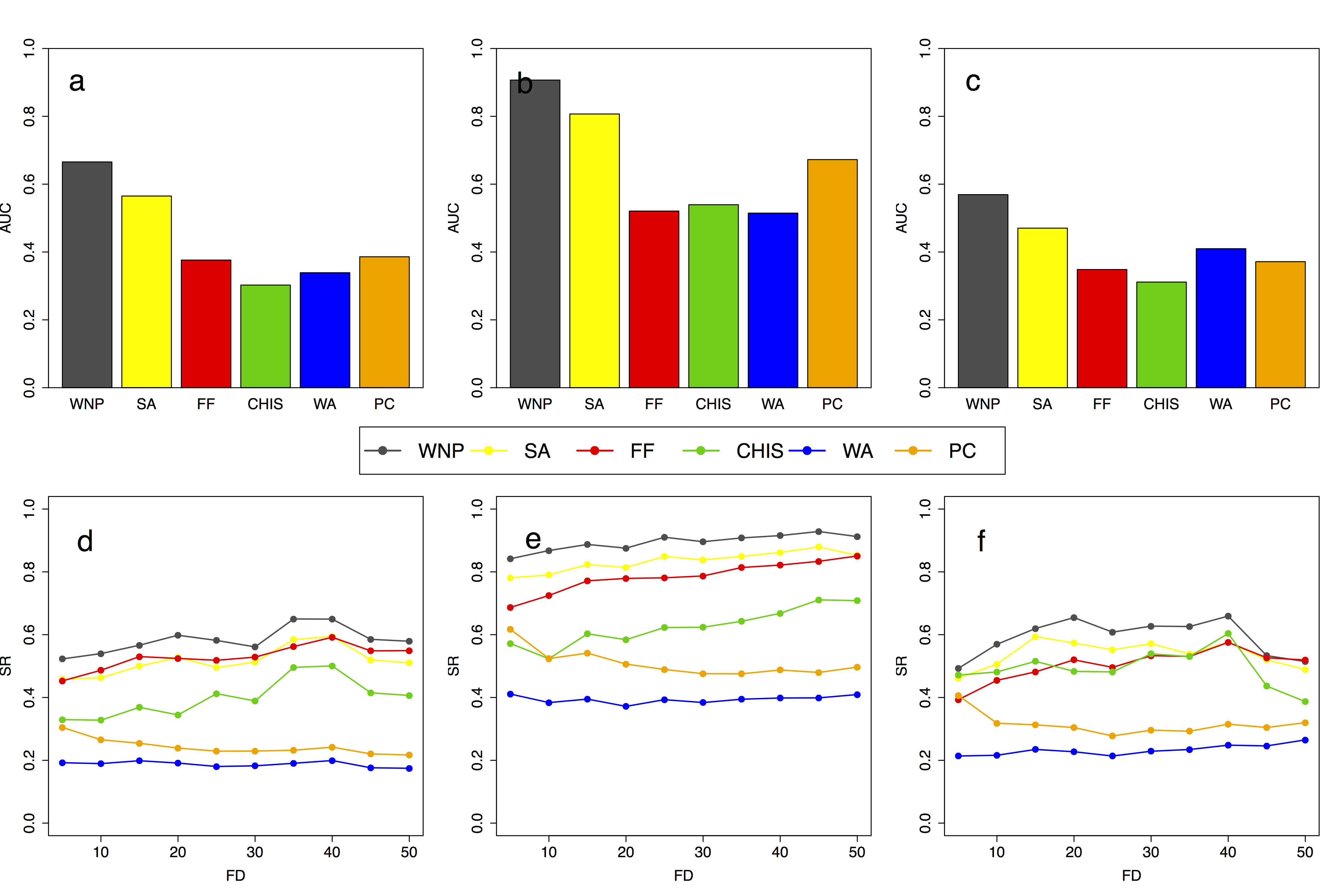

Supplement: Figure S6 — Comparison between Function prediction algorithms for Arabidopsis Thaliana. Six algorithms (WPN, SA, FF, WA, PC and CHI-Square) are compared with leave-a-percent-out criterion for 10% of annotated GPs cleared. For each algorithm the area under the ROC curve (AUC) and the FD vs. SR curves are averaged across 100 simulations. The results are reported for the three categories of the GO database: cellular component (a, d), biological process (b, e) and molecular function (c, f). (TIFF) [file pone.0038767.s006.tif]

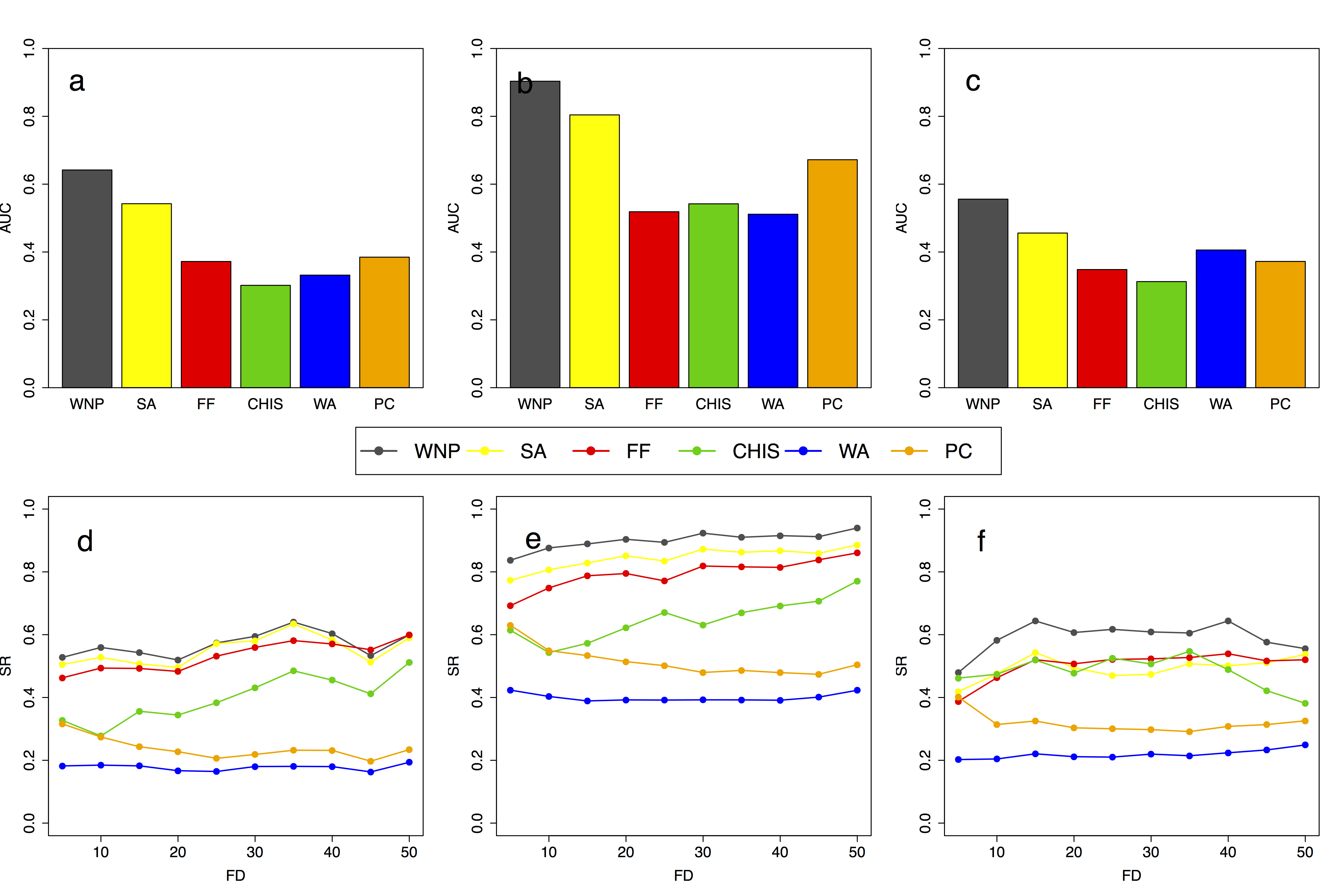

Supplement: Figure S7 — Comparison between Function prediction algorithms for Arabidopsis Thaliana. Six algorithms (WPN, SA, FF, WA, PC and CHI-Square) are compared with leave-a-percent-out criterion for 15% of annotated GPs cleared. For each algorithm the area under the ROC curve (AUC) and the FD vs. SR curves are averaged across 100 simulations. The results are reported for the three categories of the GO database: cellular component (a, d), biological process (b, e) and molecular function (c, f). (TIFF) [file pone.0038767.s007.tif]

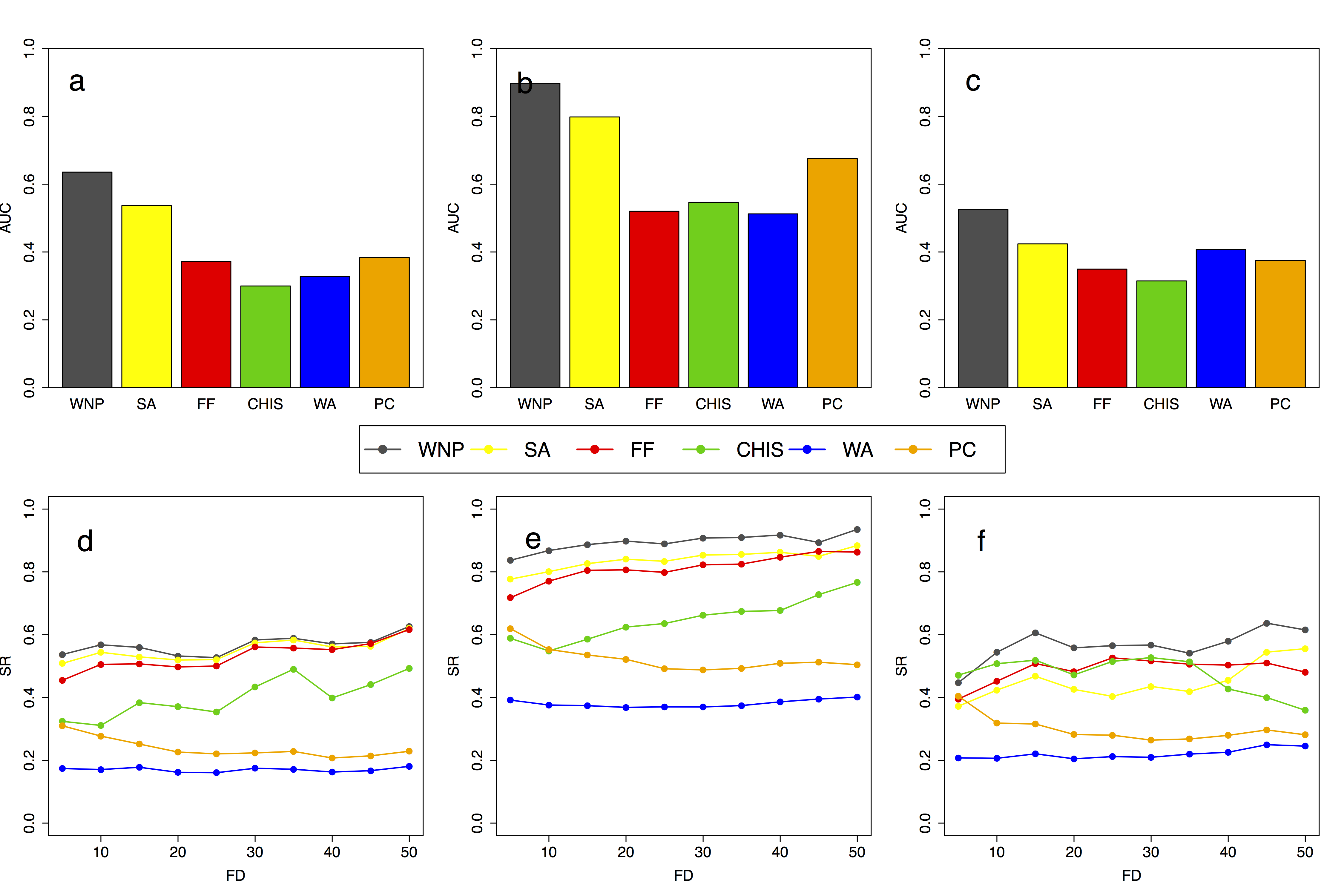

Supplement: Figure S8 — Comparison between Function prediction algorithms for Arabidopsis Thaliana. Six algorithms (WPN, SA, FF, WA, PC and CHI-Square) are compared with leave-a-percent-out criterion for 20% of annotated GPs cleared. For each algorithm the area under the ROC curve (AUC) and the FD vs. SR curves are averaged across 100 simulations. The results are reported for the three categories of the GO database: cellular component (a, d), biological process (b, e) and molecular function (c, f). (TIFF) [file pone.0038767.s008.tif]

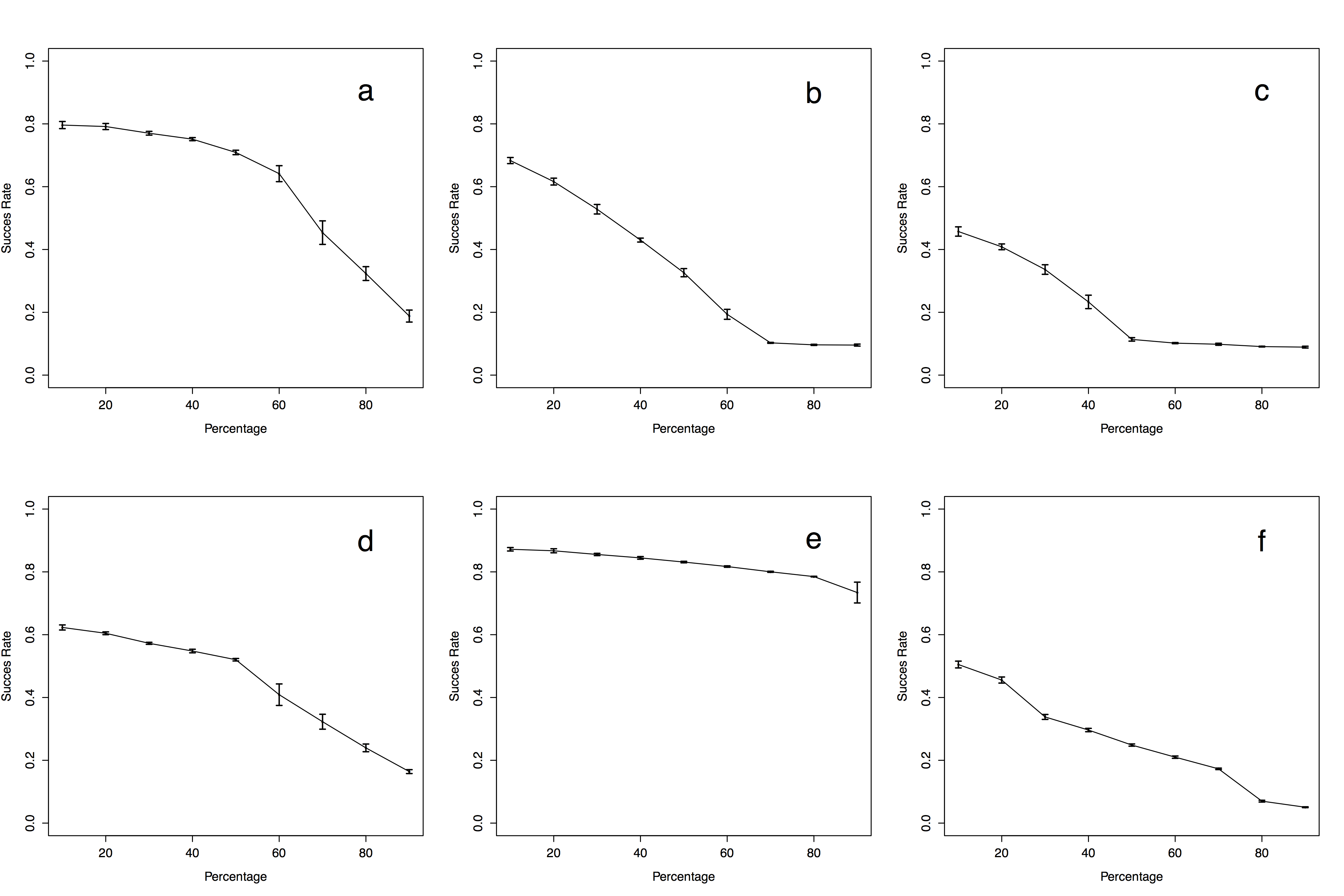

Supplement: Figure S9 — Prediction Success rate as a function of cleared GPs percentage. The prediction accuracy of WPN algorithm is tested on leave-a-percent-out datasets with cleared annotated GPs that ranges between 10% and 90%. Each point represent the mean value of success rate across 100 simulations, while error bars are the standard deviation. The leave-a-percent-out validations were performed for Saccharomyces Cereviasiae (a, b, c) and Arabidopsis Thaliana (d, e, f). The results are reported for the three categories of the GO database: cellular component (a, d), biological process (b, e) and molecular function (c, f). (TIFF) [file pone.0038767.s009.tif]
