# Supplementary material for: WNP: A Novel Algorithm for Gene Products Annotation from Weighted Functional Networks
Source: PLoS One. 2012 Jun 28;7(6):e38767. doi: 10.1371/journal.pone.0038767 (PMC3386258; doi:10.1371/journal.pone.0038767)
Supplement: Text S1 — Details concerning methods discussed in this work. (PDF) [file pone.0038767.s012.pdf]

Supplementary Material to "WNP: a novel algorithm for gene products annotation from weighted functional networks."

Alberto Magi, Lorenzo Tattini, Matteo Benelli, Betti Giusti, Rosanna Abbate and Stefano Ruffo.

Supplementary Methods

Generalized Simulated Annealing

In order to minimize the Weighted Score E_w, we used the Generalized Simulated Annealing (GSA) introduced by Tsallis and Stariolo, [1], instead of the classical Simulated Annealing (SA) [4] used by Vazquez and coworkers [2]. SA is a general-purpose stochastic optimization technique, based on the Metropolis-Hastings algorithm [3]. In GSA the acceptance probability P_E is based on Tsallis statistics instead of Boltzman distribution:

$$P_E = [1 - (1-q)\beta\Delta E]^{1/(1-q)} \tag{1}$$

where ΔE is the change in the potential energy, $\beta = 1/kT$ and q is a free parameter. The important feature of Tsallis generalized statistic for optimization problems is that the probability of states does no longer decrease exponentially with energy but according to a power law where the exponent is determined by the free parameter q. The parameter q is varied as a monotonically decreasing function of temperature $q(T)$. Starting with a convenient value of q at the initial temperature, q tends towards 1 as the temperature decreases during annealing [5]. The algorithm is initialized with a configuration of states $\sigma = [\sigma_1, \sigma_2, ..., \sigma_n]$ selected among the possible biological functions and randomly assigned to uncharacterized GPs. At each step of the Monte Carlo simulation we choose a random unclassified GP i and substitute his state σ_i with a new state σ_i', where σ_i' is randomly selected among the possible biological function with the constraint $\sigma_i \neq \sigma_i'$, as suggested in Ref. [2]. After every function substitution we calculated the total energy $E(\sigma_i)$ and $E(\sigma_i')$ for both the new and old states: if the difference $\Delta E = E(\sigma_i) - E(\sigma_i')$ is minor than 0 the new state is accepted. If ΔE is equal or greater than 0 the new state is accepted with probability:

$$min(1, [1 - (1-q)\beta\Delta E]^{1/(1-q)}) \tag{2}$$

To set up the initial tempereture T_0 of the simulated annealing schedule we generated 1000 random solutions E_{0i} and we took T_0 as the standard deviation of these solutions:

$$T_0 = \sqrt{\frac{1}{N}\sum_{i=1}^{N}(E_{0i} - \overline{E0})} \tag{3}$$

where $\overline{E0}$ is the mean of E_{0i} and N=1000.

Starting from an initial temperature T_0 at which nearly all transitions are accepted, we used an exponential temperature decreasing scheme:

$$T_{k+1} = aT_k \tag{4}$$

with $a = 0.99$. We choose the length of the Markov process independently for each performed simulation, with the constrain that the proportion of accepted states in the whole minimization process ranges between 0.4 and 0.6. The minimization process is stopped when two consecutive iterations give the same solutions. At the end of the minimization process a biological function is assigned to each uncharacterized gene product (GP) and these are the predicted classification. However, since the minimum energy solution is not unique or the optimization technique can be entrapped in a local minimum, we repeat the simulated annealing several times (100 simulations) starting from different initial configuration. At the end of the simulation we calculate the fraction of time (p_i) the GP i has been predicted to have function σ_i, and this is the probability that the GP i belong to the functional classification σ_i.

Algorithms Comparison

We compared the prediction capability of WNP algorithm with other five state-of-the-art methods: Simulated Annealing (SA) approach by [2], FunctionalFlow (FF) [6], ChiSquare (CHIS) [7], the FS Weighted Averaging (WA) [8] and the weighted average scheme (PC) proposed Ref. [9]. SA algorithm was implemented in our labs in Fortran language following the recipe of Vazquez *et al.*. FF algorithm was implemented in our labs as an R script and all the simulations were performed by using a number of iterations $d = 6$ as suggested in Ref. [6]. The simulations for ChiSquare, WA and PC algorithms were run by using the FSWeight perl package Version 2.2 implemented by Chua Hon Nian and downloaded from `http://www.comp.nus.edu.sg/~wongls/projects/functionprediction/fsweight-15may08/`.

References

[1] Tsallis, C. and Stariolo, D.A. (1996) Generalized simulated annealing. *Physica A*, **233**, 395-406.

[2] Vazquez,A., Flammini,A., Maritan,A., Vespignani,A. (2003) Global protein function prediction from protein-protein interaction networks. *Nat Biotechnol.* **21**, 697-700.

[3] Metropolis, N., Rosenbluth, A.W., Rosenbluth,M.N., A. Teller,A.H, Teller, E. (1953) Equation of State Calculation by Fast Computing Machines. *J. Chem. Phys.* **21**, 1087-1093.

[4] Kirkpatrick,S., Gellat,C.D. and Vecchi,M.P. (1983) Optimization by simulated annealing. *Science* **22**, 671-680.

[5] Andricioaei,I. and Straub,J.E. (1996) Generalized simulated annealing algorithms using Tsallis statistics: Application to conformational optimization of a tetrapeptide. *Physical Review E* **53**, 3055-5058.

[6] Nabieva,E., Jim,K., Agarwal,A., Chazelle,B., Singh,M. (2005) Whole-proteome prediction of protein function via graph-theoretic analysis of interaction maps. *Bioinformatics* **21** (Suppl 1), i302-i310.

[7] Hishigaki,H., Nakai,K., Ono,T., Tanigami,A., Takagi,T. (2001) Assessment of prediction accuracy of protein function from protein-protein interaction data. *Yeast* **18**, 523-531

[8] Chua,H.N., Sung,W.K., Wong,L. (2006) Exploiting indirect neighbours and topological weight to predict protein function from protein-protein interactions. *Bioinformatics* **22**, 1623-1630

[9] Chua,H.N., Sung,W.K., Wong,L.v(2007) An efficient strategy for extensive integration of diverse biological data for protein function prediction. *Bioinformatics* **23**, 3364-3373.
